# Supplementary material for: Triggering typical nemaline myopathy with compound heterozygous nebulin mutations reveals myofilament structural changes as pathomechanism
Source: Nat Commun. 2020 Jun 1;11:2699. doi: 10.1038/s41467-020-16526-9 (PMC7264197; doi:10.1038/s41467-020-16526-9)

Triggering typical nemaline myopathy with compound heterozygous nebulin mutations reveals myofilament structural changes as pathomechanism.

Supplementary Information including full lane gel images

Lindqvist et al.

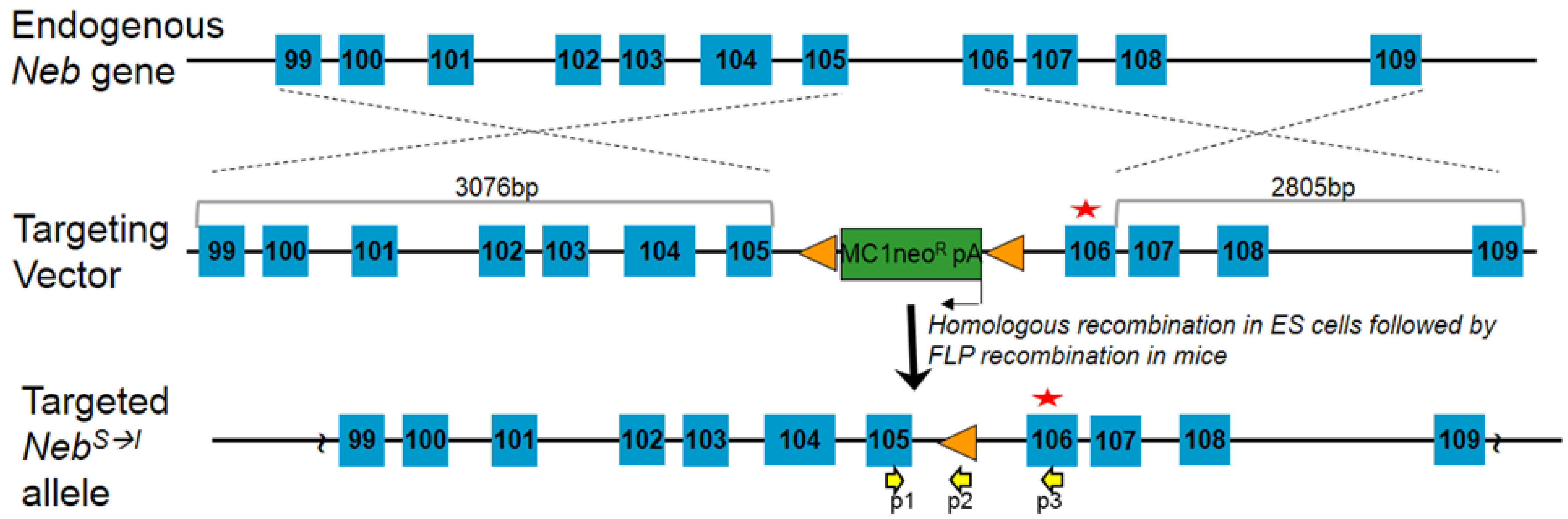

Supplementary Fig. 1. Generation of Neb^{S6366I} and Compound-Het mice. **Neb^{S6366I} mice:** Mice were generated by the University of Arizona GEMM Core facility. A targeting vector was designed to Knock-In the equivalent NM_001271208.2(NEB):c.19097G>T (p.Ser6366Ile) missense variant into mouse (in exon 106 of Ensembl transcript ENSMUST00000238749.1) using the Osdd vector which provides TK as a negative selection cassette outside the targeting region. Overlapping primers introduced two nucleotide changes, introducing an MfeI restriction site (to facilitate distinguishing the targeted Knock-in and endogenous transcripts as well as allowing design of allele-specific PCR primers) and resulting in an S→I missense variant. Linearized targeting vector was electroporated into 129S6/SvEvTac cells; 6 G418-resistant ES cell clones were confirmed to have correctly integrated into the *Neb* gene. Two of these clones were injected into blastocysts, transferred into host mothers, and produced chimeras. Both clones went germline; no differences were observed between mice from the 2 clones; only mice from clone #166 were used in this study. Mice were crossed to FlpO (B6.Cg-Tg(Pgk1-flpo)10Sykr/J, #11065 Jackson Laboratories) to remove the *neo*-cassette. A single Het female was back-crossed to a Black6 (C57BL/6J, #664 Jackson Laboratories) male for 10 generations to establish the strain. Genotyping primers used: P1, 5'-ATCGTCATCTTGGCTTGGTT-3'; P2, 5'-CTGGCACTCTGTCGATACCC-3'; P3, 5'-TGTCTTTTTCCCTCCAAACG-3'. **Generation of Compound-Het mice**. Compound heterozygous Neb$^{S6366I,\Delta exon55}$ mice were produced by breeding Neb^{S6366I} Het mice with Neb$^{\Delta exon55}$ Het mice (MGI allele Nebtm1.1Hgra #5553119) (Ottenheijm et al 2013), which had also had the neo cassette removed and been backcrossed to C57BL/6J for 10 generations.

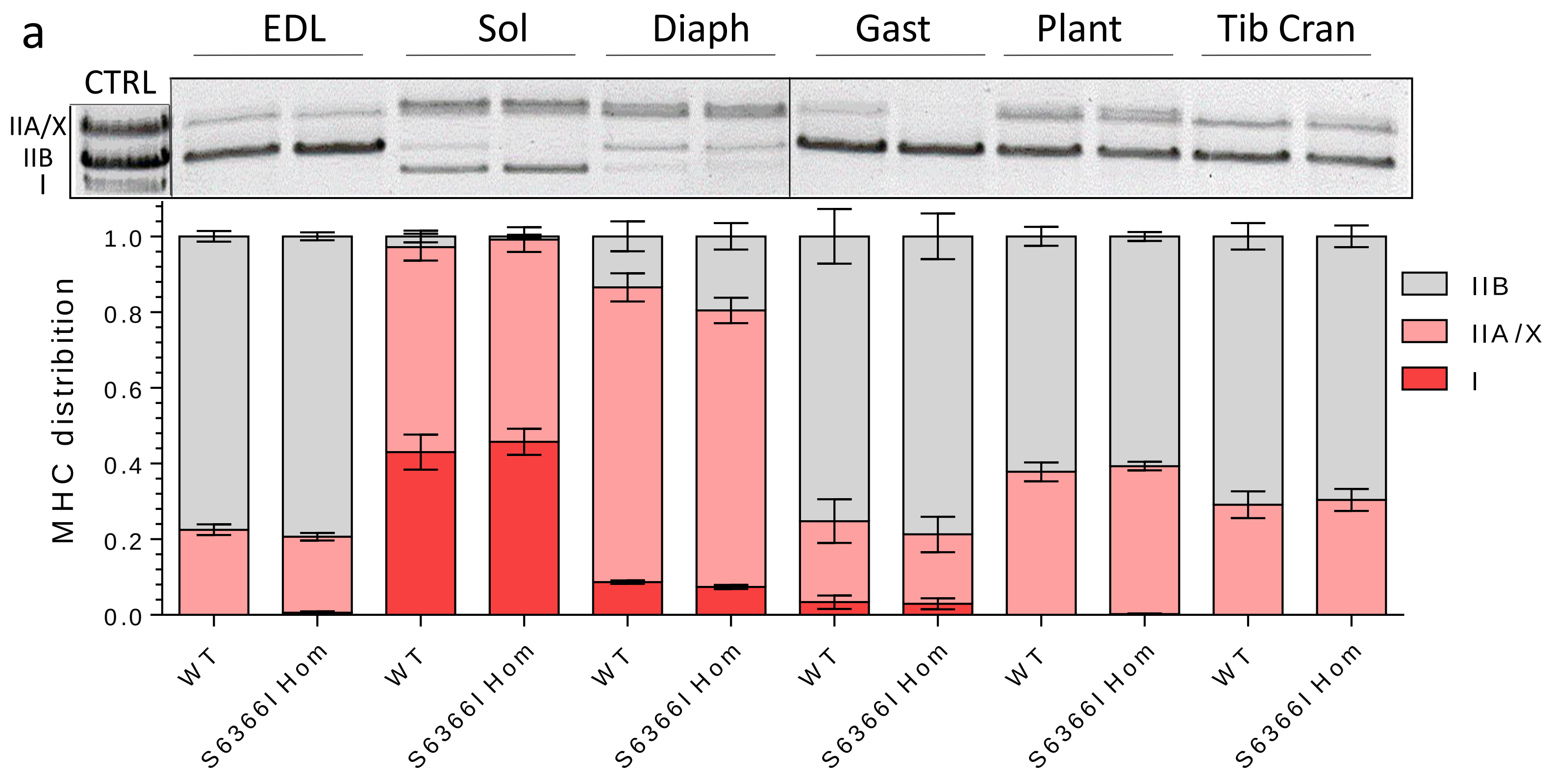

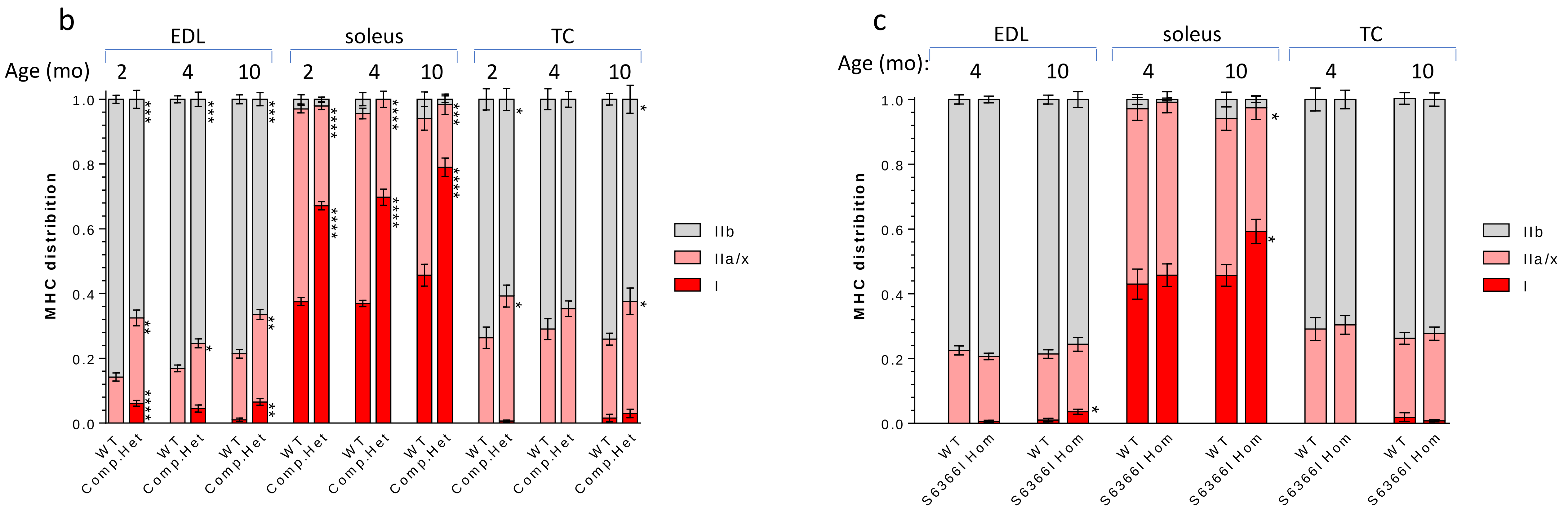

Supplementary Fig. 2. Myosin heavy chain (MHC) isoform expression in Neb^{S6366I} Hom and Compound-Het mice.

a) Top representative MHC gel images; bottom analyzed results. The controls (CTRL) are pooled homogenates of TC and Soleus muscle, revealing the 3 isoforms that can be discerned: type I, IIA/X and IIB (from bottom to top). No changes occur in MHC expression in any of the muscle types. Number of mice WT: 6, and S6366I Hom: 8.

b) Comparison of MHC distribution in 2, 4, and 10 mo old mice in EDL, soleus and TC muscles of Compound-Het mice. n(2 mo-WT)=6 mice; n(2 mo-Compound-Het)= 6 mice; n(4 mo-WT)=7 mice; n(40-Compound-Het)=9 mice; n(10 mo- WT)= 6 mice; n(10 mo-Compound-Het)=6 mice.

c) Comparison of MHC distribution in EDL, soleus and TC muscles of 4 mo and 10 mo old S6366I mice. n(4 mo-WT)=6 mice; n(4mo-Compound Het)= 8 mice; n(10 mo-WT)= 6 mice; n (10 mo-Compound Het)= 6 mice.

a-c: Two-way ANOVA with no matching and a posthoc multiple testing comparison with multiple testing correction (Sidak). Values are mean ± SEM. *p<0.05; **p<0.01; ***p<0.001; ****p<0.0001.

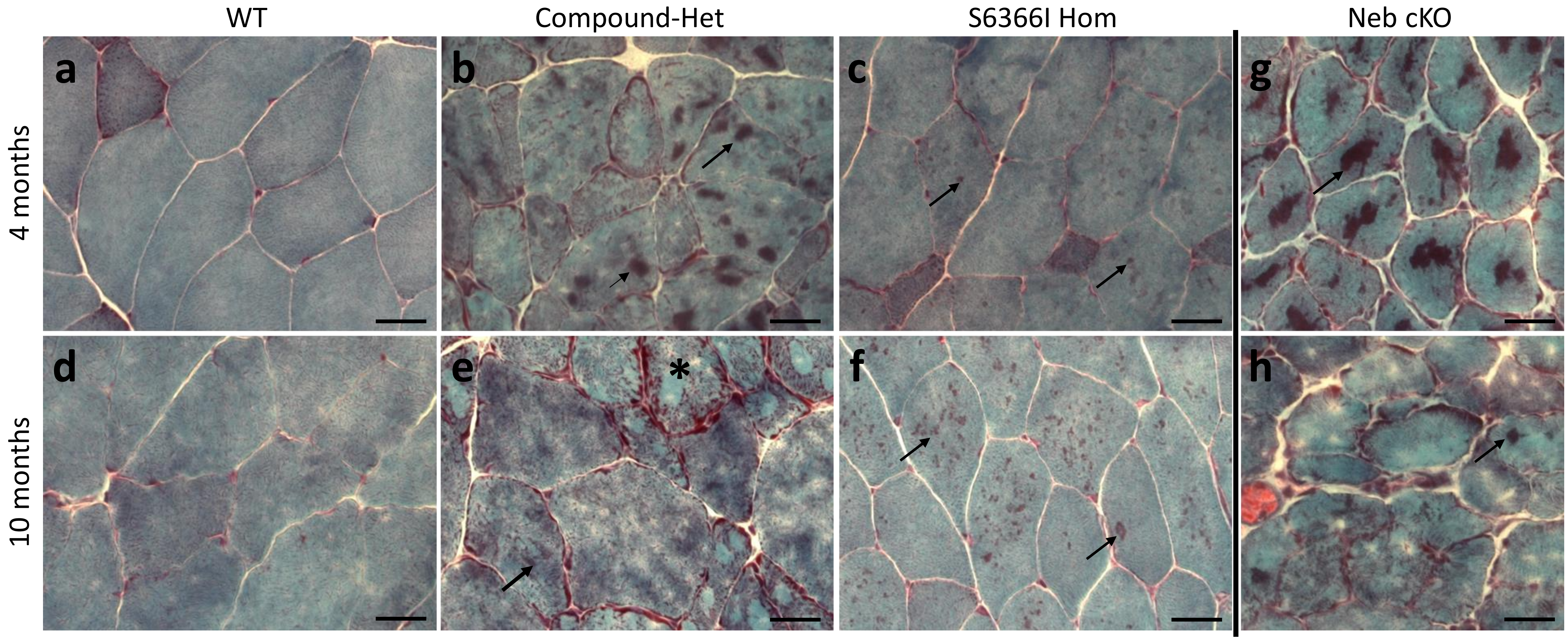

Supplementary Fig. 3 Gomori trichrome stained gastrocnemius muscles.

Histological findings in gastrocnemius muscles of Compound-Het, S6366I Hom and WT mice at 4 mo (a-c) and 10 mon (d-f). Nemaline rods (arrows) are visible on modified Gomori trichrome stained sections of both Compound-Het and S6366I Hom mice. Core-like structures are visible in 10-months old Compound-Hets (asterisk). g and h) Nemaline rod bodies in soleus (g) and EDL (h) muscles from conditional nebulin KO mice with severe nemaline myopathy as comparison. 2 mice were studied for each of the 8 groups, with identicalresults. Scale bar = 20 µm.

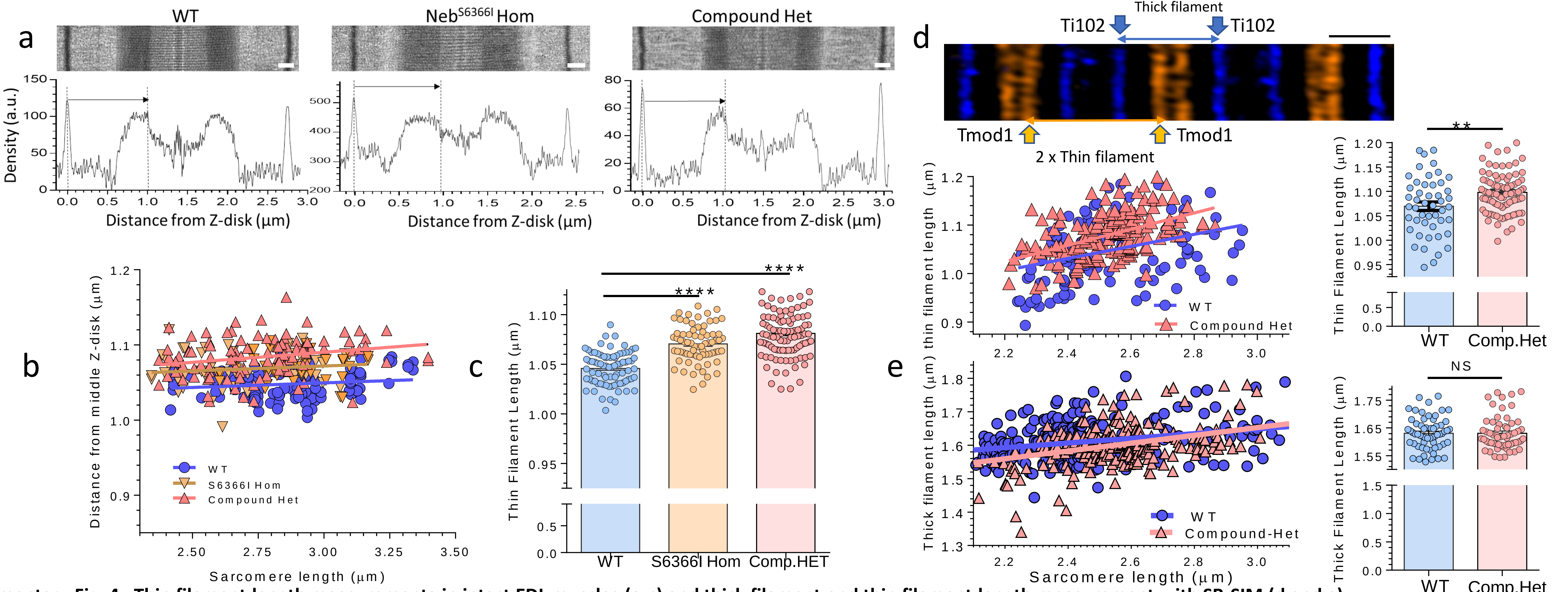

Supplementary Fig. 4. Thin filament length measurements in intact EDL muscles (a-c) and thick filament and thin filament length measurement with SR-SIM (d and e).

a) Top: Representative electron-micrographs of sarcomeres in intact EDL muscle ; bottom: densitometry indicating how thin filament lengths were measured (arrows). Obtained thin filament length values were corrected for shrinkage obtained by comparing measured A-band width to the *in vivo* thick filament length of 1.6 µm (shrinkage was typically ~5%). Scale bar: 200 µm. Studied were 81 sarcomeres from 18 fibers from 6 WT mice, 71 sarcomeres from 18 fibers from 6 Compound-Het mice and 103 sarcomeres from 18 fibers from 6 S6366I Hom mice.

b) Thin filament length vs sarcomere length graphs. Linear regression analysis with as null-hypothesis: 1. The slopes are equal. Test reveals that the null hypothesis can not be rejected (p=0.39; F= 0.94, DFn=2, DFd=332). 2. Are the elevations or intercepts equal? This null hypothesis can be rejected (p=<0.0001; F = 96.057. DFn=2 DFd=334).

c) Mean ± SEM of thin filament length in SL 2.5-3.0 µm range. ANOVA with multiple-testing correction (Tukey, no matching or pairing) reveals significantly longer thin filaments in S6366I HOM (24 nm longer than WT) and Compound-Het EDL (41 nm longer than WT). n:(WT)=81 measurements from 18 fibers from 6 mice; n (Hom)=71 measurements from 18 fibers from 6 mice; n (Compound HET)=103 measurements from 18 fibers from 6 mice. ****p<0.0001.

d and e) Thin filament (d) and thick filament (e) length measurements by SR-SIM microscopy in EDL muscle from WT and Compound-Het mice (4 mo old, 3 each). d) Top, example image showing Tmod1 labeling (at pointed end of thin filament) and Ti102 labeling (at edge of thick filament) Scale bar: 1.0 µm; d Left Bottom) thin filament length (distance between Tmod1 and Tmod1, as indicated on image, divided by 2) vs sarcomere length graph; d Bottom right) mean ± SEM of thin filament length in the SL 2.5-3.0 µm range. In Compound-Het thin filaments are significantly longer than in WT mice (by 29 nm). Unpaired two-tailed T-test, **p=0.0017. E left) thick filament length (Ti102-Ti102 distance, as indicated on image) vs. SL; e Right) Mean ± SEM of thick filament length data in the SL 2.5-3.0 µm range. Thick filament lengths are not different. Two-tailed unpaired t-test. For thick filament: p=0.98. ns: non-significant. n(WT)=48 thin-filament length measurements from 15 fibers from 3 mice; n(Compound-Het)= 91 thin-filament measurements from 15 fibers from 3 mice; n(WT)=60 thick-filament length measurements from 5 fibers from 3 mice; n(Compound-Het)=61 thick-filament length measurements from 15 fibers from 3 mice.

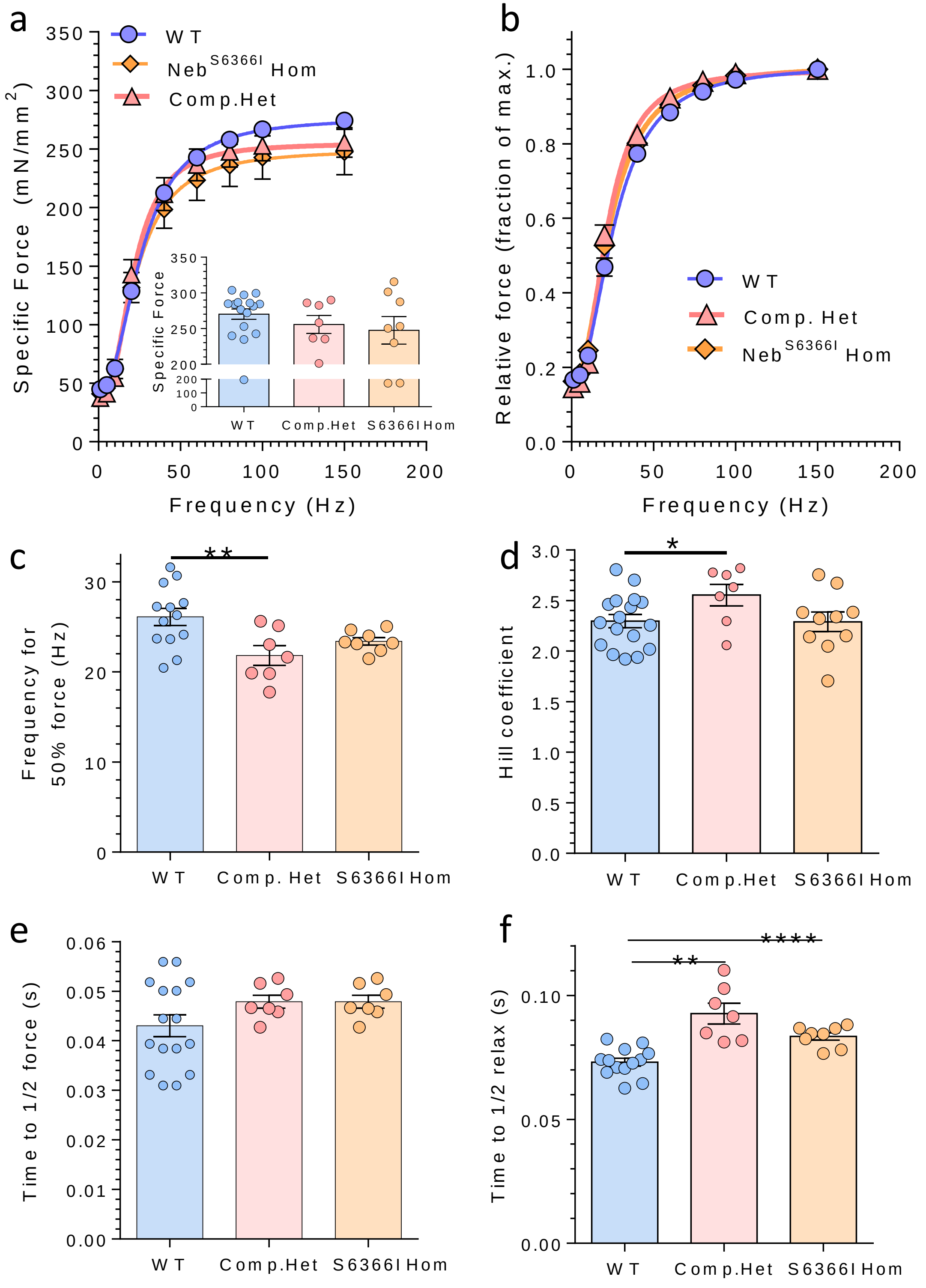

Supplementary Fig. 5 Intact whole Soleus muscle mechanics.

a) Force-frequency relation of WT, Compound-Het and S6366I Hom soleus muscle. Inset shows maximal force (150 Hz stimulation rate). Force is unaltered in Compound-Het and S6366I Hom mice. (Force expressed as specific force: force per unit area of muscle, mN/mm^2).

b) Relative force vs stimulation frequency is slightly steeper in Compound-Het mice.

c) Frequency for 50% force is reduced in Compound-Het mice. **p=0.007

d) Hill coefficient is increased in Compound-Het mice. *p=0.049

e) Time to ½ maximal force of 150 Hz tetanus is unaltered.

f) Time to ½ relaxation of 150 Hz tetanus is longer in Compound-Het mice and Neb^{S6366I} HOM mice. ****p<0.0001, **p=0.007

a and b) Non-linear least squares fit to Hill curve with as null-hypothesis that one curve fits all data sets. Test reveals that the null hypothesis is rejected in each panel (p=0.04 in a and p<0.0001 in b). a inset and c-f) Ordinary one-way ANOVA without matching or paring. A posthoc multiple testing comparison with multiple testing correction (Tukey) was performed. Number of mice: WT: 13, Compound-Het: 7, S6366I Hom: 8. Values are means ± SEM.

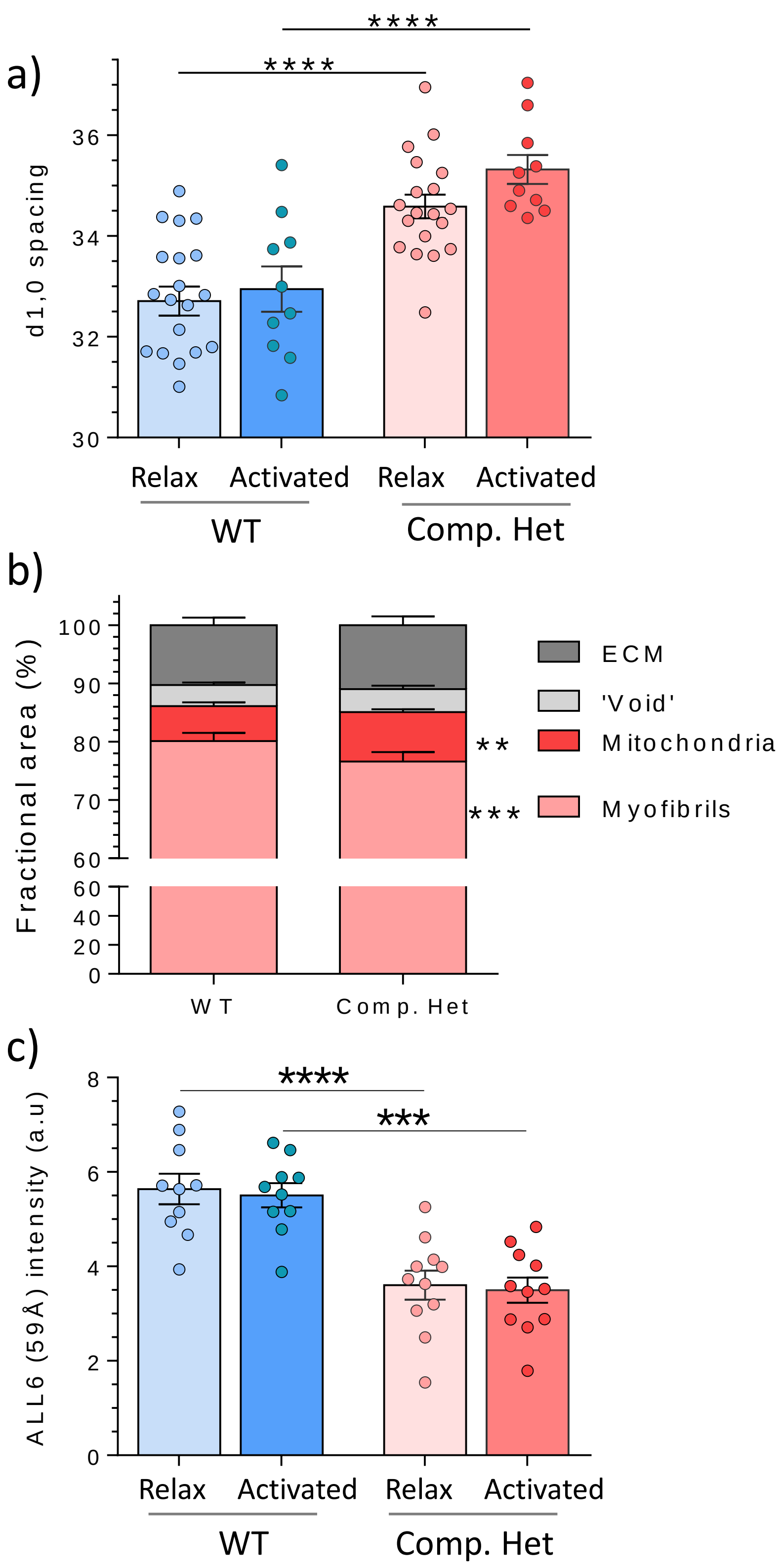

Supplementary Fig. 6 Spacing of the 1,0 equatorial reflections (d1,0) and its conversion into number of thin filaments per mm^2 of cross-sectional area muscle. Mean values during activation (d1,0 WT: 32.95 nm; Compound-Het 35.32 nm) were used to calculate the area of the unit cell in activated muscle: $2/(sqrt3) * d_{10}^2$. Considering that a unit cell contains two thin filaments, this results in 1.594 x 10^9 thin filament per mm^2 myofibril in WT and 1.389 x 10^9 thin filament per mm^2 myofibril in Compound-Het (or 12.9% less). Accounting for the myofibrillar fractional area of 0.801 in WT and 0.766 in Compound-Het (see below) results in 1.2943 x 10^9 thin filament per mm^2 muscle in WT and 1.0644 x 10^9 thin filament per mm^2 muscle in Compound-Het. These values make it possible to calculate the force per thin filament using the specific tetanic forces measured in muscle. However, the force gradient along the thin filament has to be taken into account as well (zero force at the H-zone, maximal force from A-band edge to Z-disk). Assuming maximal filament overlap during contraction and a thin filament length of 1.05 µm in WT and 1.08 µm in Compound-Het (Supplemental Figure 4c), and 0.03 um of the thin filament incorporated into the Z-disk (from edge of Z-disk to middle of the Z-disk), results in 0.295 µm I-band filament with 100% force in WT and 0.326 µm I-band filament with 100% force in Compound-Het sarcomere. The thin filament length with overlap is 0.725 µm in both genotypes. This results in an average force along the thin filament of 65.75% of maximal force in WT and 68.85% force in Compound-HET. Number of mice: WT: 15, Compound-HET: 11. For some animals both EDL muscles were used. Number of muscles: WT: 19, Compound-HET: 19. Activated muscles: 10 per group. Unpaired two-tailed T-tests between WT and Compound-Het in relax and activated condition, respectively. ****p<0.0001.

b) Fractional cross-sectional area of myofibrils is reduced in Compound-Het EDL muscle (from 0.812 to 0.7663) and mitochondrial area is increased (from 0.06 to 0.0847). Void area is defined as sarcotubular system, vacuoles, and lipid droplets, as well as rod bodies. Results from 5 WT and 6 Compound-Het mice. Lines are mean ± SEM. Two-way ANOVA with no matching and a posthoc multiple testing comparison with multiple testing correction (Sidak). **p=0.002, ***p=0.0006).

c) Intensity distribution of ALL6 (59Å) in passive and active WT and Compound-Het muscle.
Lines are means ± SEM. a and c) Ordinary one-way ANOVA without matching or paring. A posthoc multiple testing comparison with multiple testing correction (Tukey) was performed. **** p<0.0001, ***p<0.001. Values are mean ± SEM. n(WT)= 8 mice; n(Compound-HET)= 8 mice. For some animals both EDL muscles were used. n(WT)=9 muscles; n(Compound-HET)=10 muscles.

Figure 3a. Uncropped gel images of nebulin isoforms.
Boxes indicate areas shown in figure.

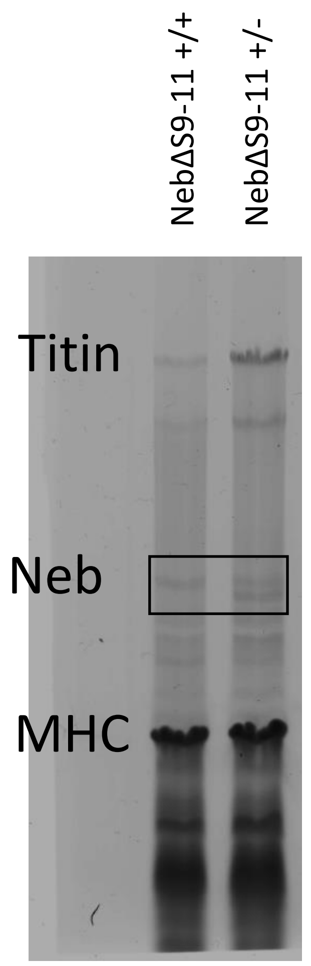

Figure 3a. Left panel

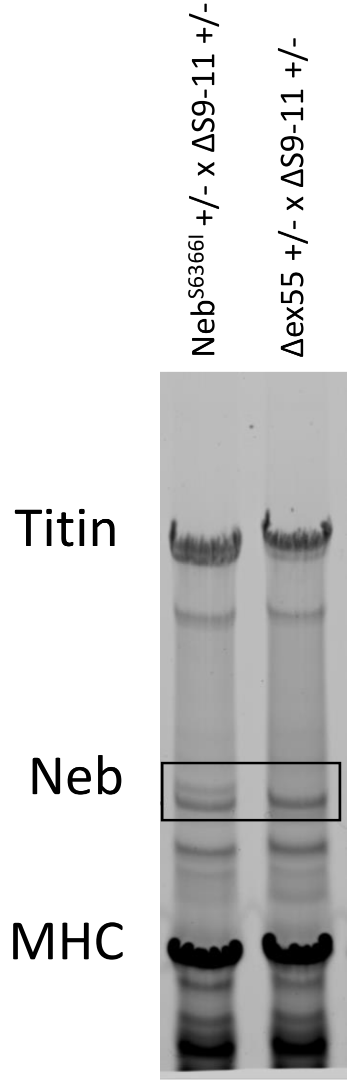

Figure 3a. Right panel

Figure 3b. Uncropped gel image.

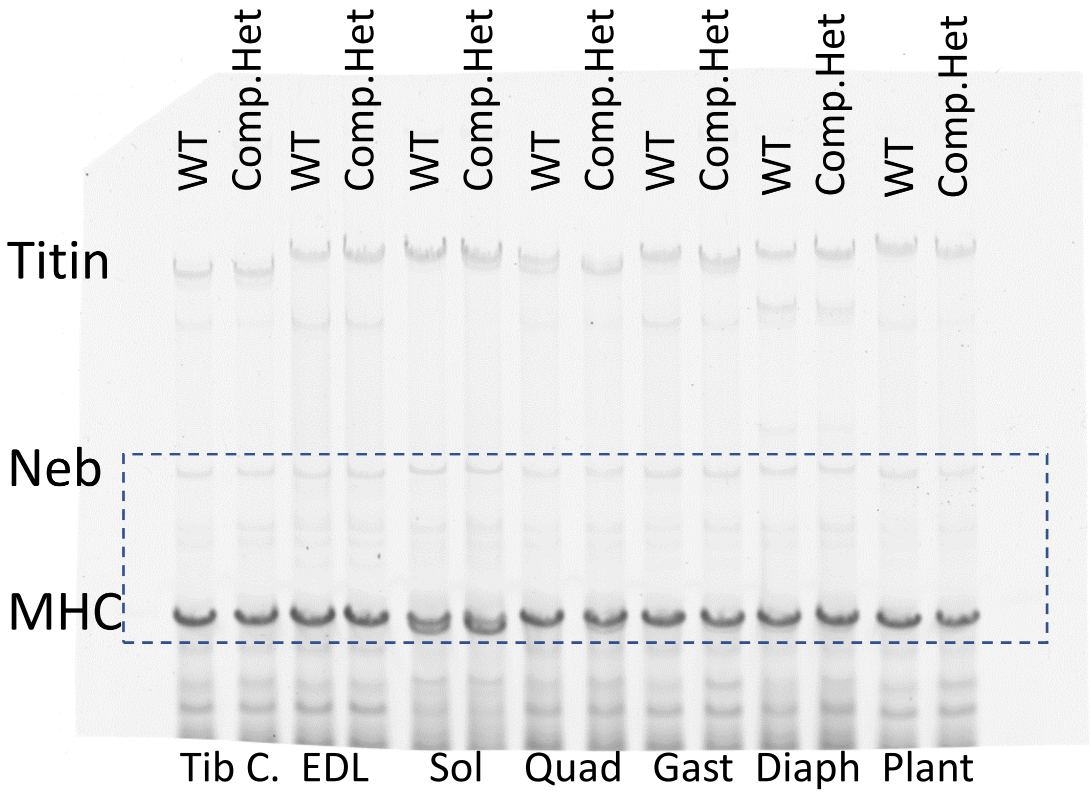

Figure 3c. Uncropped gel image.

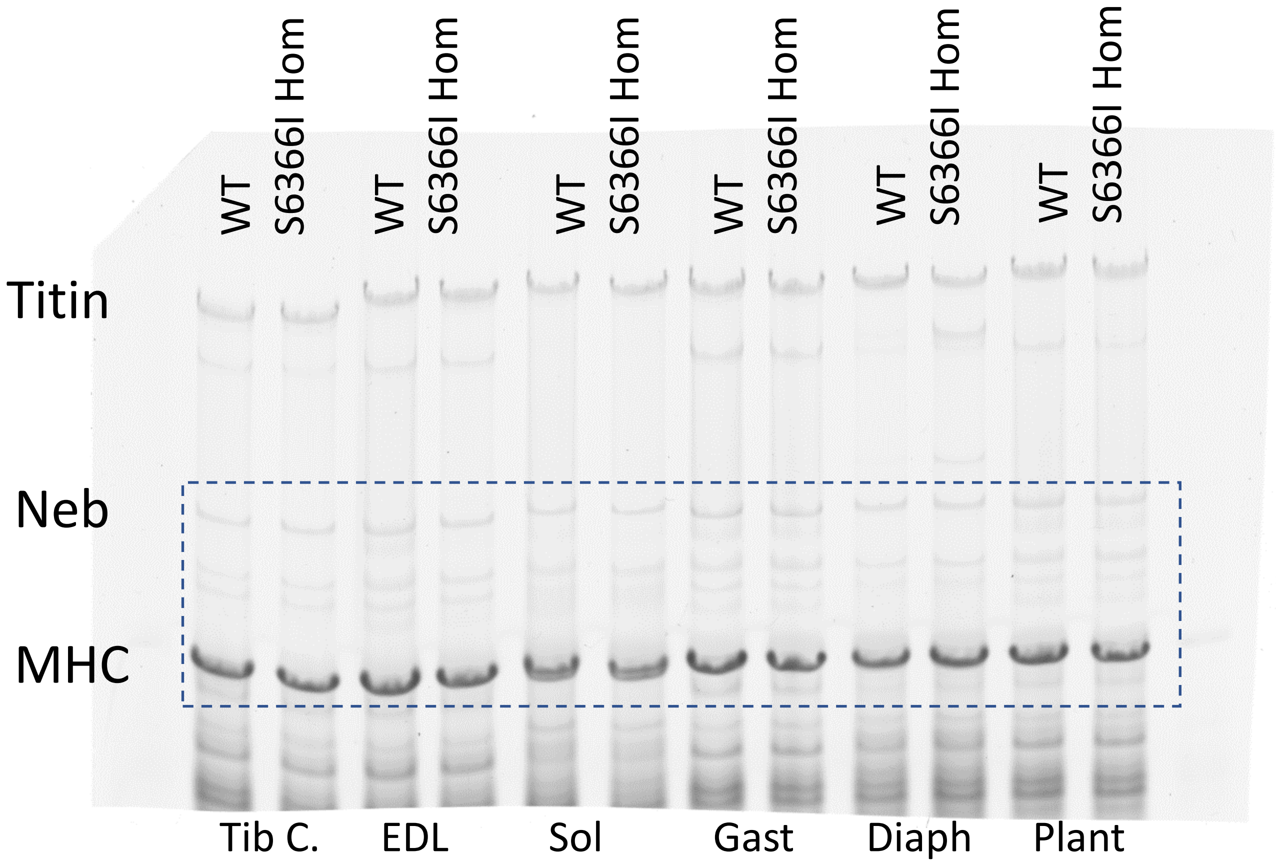

Boxes indicate areas shown in figure.

Figure 4a top. Uncropped gel image.

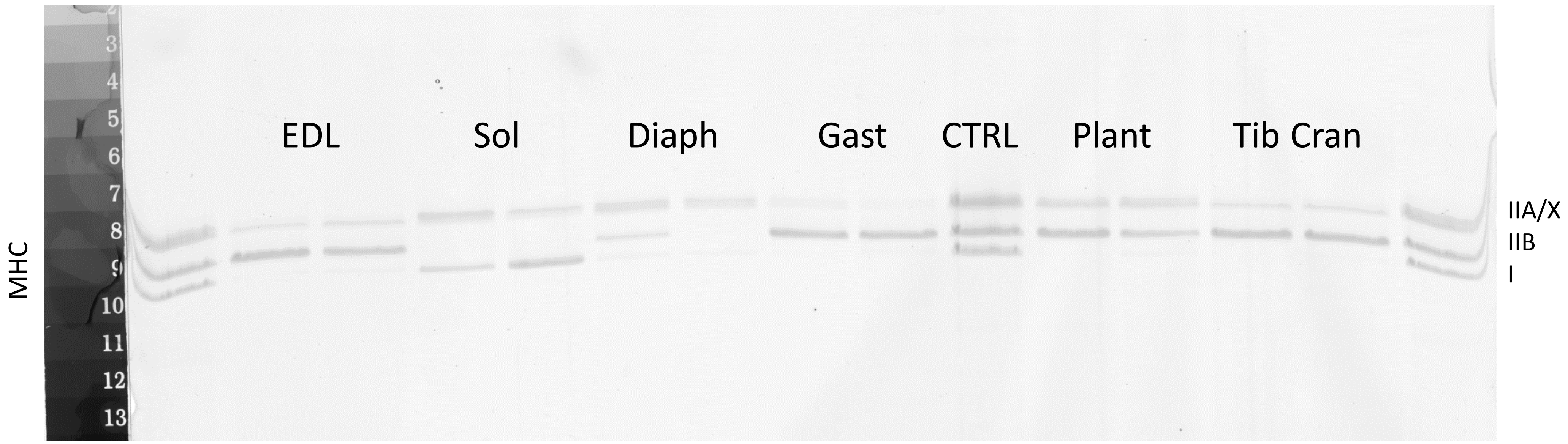

Figure 7a inset. Full scan gel image. Boxes show displayed cropped parts of inset. STD, standard.

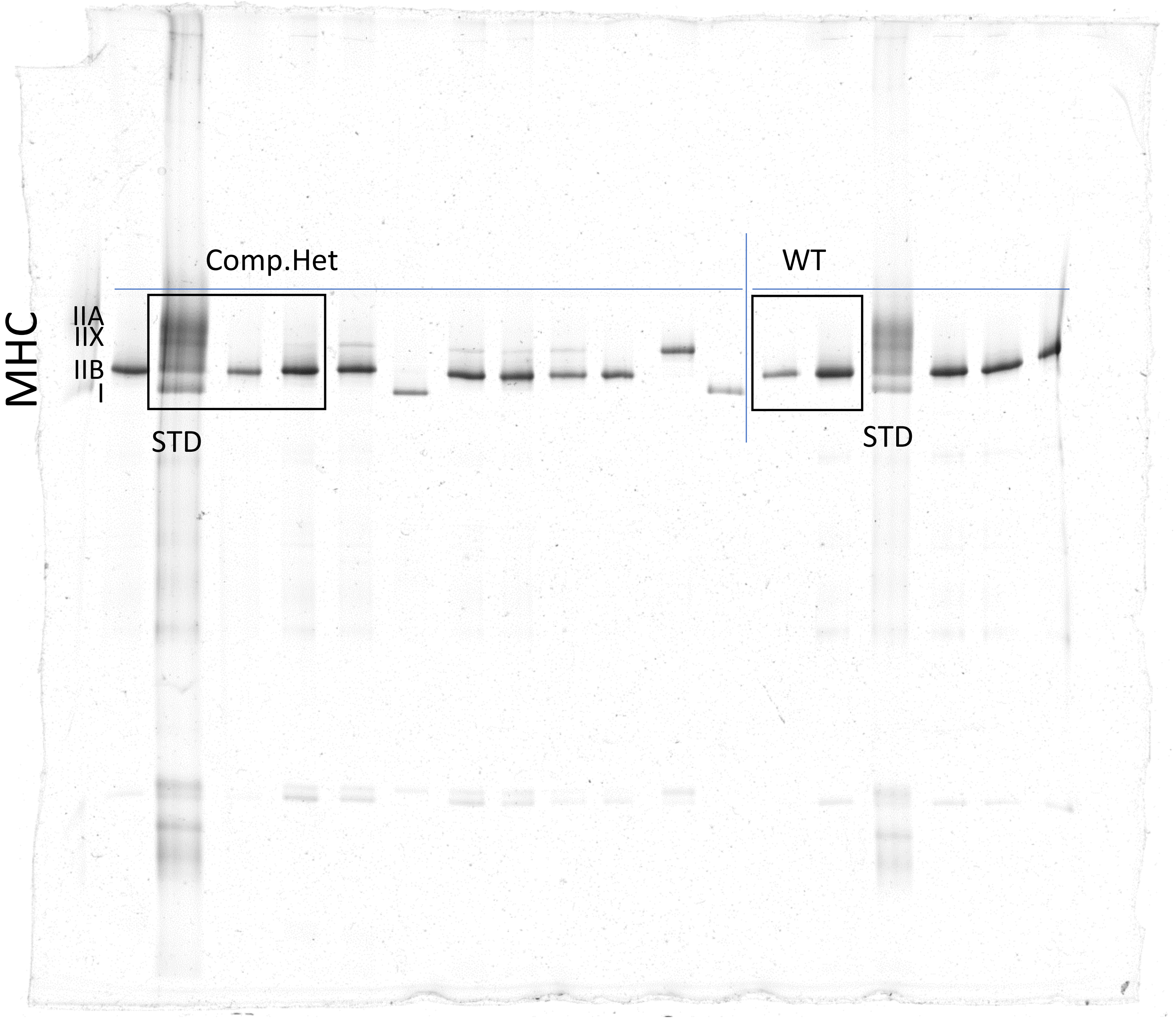

Figure S2a top. Uncropped gel image.

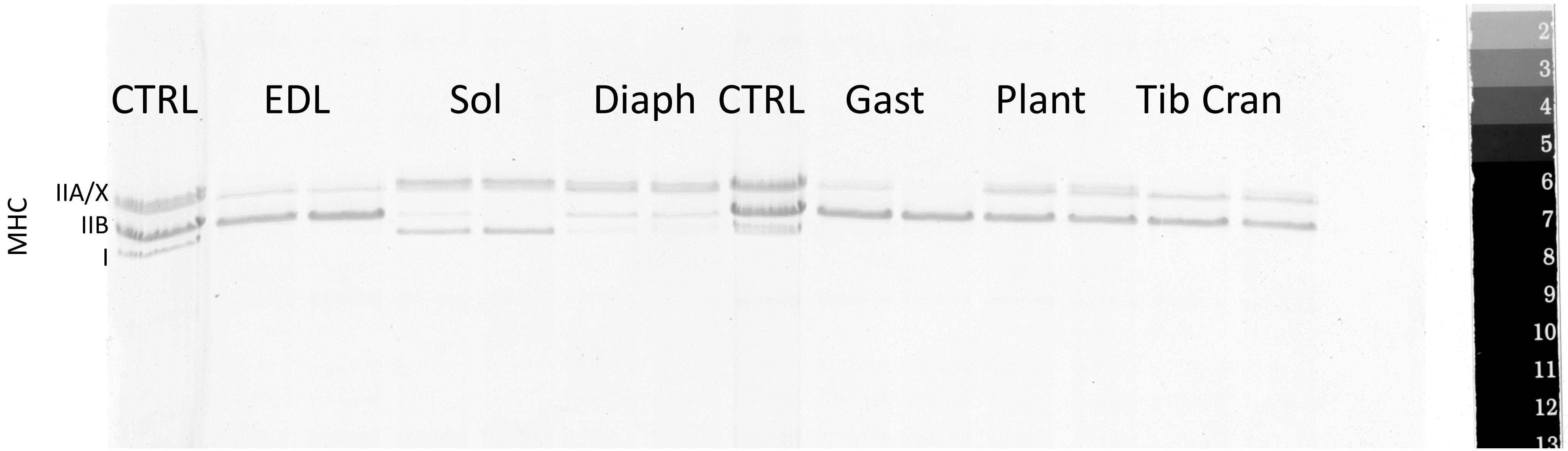

Supplement: Supplementary file 1 — Supplementary Information [file 41467_2020_16526_MOESM1_ESM.pdf]
