# Supplementary material for: Triggering typical nemaline myopathy with compound heterozygous nebulin mutations reveals myofilament structural changes as pathomechanism
Source: Nat Commun. 2020 Jun 1;11:2699. doi: 10.1038/s41467-020-16526-9 (PMC7264197; doi:10.1038/s41467-020-16526-9)
Supplement: Supplementary file 2 — Reporting Summary [file 41467_2020_16526_MOESM2_ESM.pdf]

nature research

Corresponding author(s): Dr. Henk L. Granzier

Last updated by author(s): Apr 4, 2020

Reporting Summary

Nature Research wishes to improve the reproducibility of the work that we publish. This form provides structure for consistency and transparency in reporting. For further information on Nature Research policies, seeAuthors & Referees and theEditorial Policy Checklist .

Statistics

For all statistical analyses, confirm that the following items are present in the figure legend, table legend, main text, or Methods section.

n/a	Confirmed	
[ ]	[x]	The exact sample size (*n*) for each experimental group/condition, given as a discrete number and unit of measurement
[ ]	[x]	A statement on whether measurements were taken from distinct samples or whether the same sample was measured repeatedly
[ ]	[x]	The statistical test(s) used AND whether they are one- or two-sided<br>*Only common tests should be described solely by name; describe more complex techniques in the Methods section.*
[ ]	[x]	A description of all covariates tested
[ ]	[x]	A description of any assumptions or corrections, such as tests of normality and adjustment for multiple comparisons
[ ]	[x]	A full description of the statistical parameters including central tendency (e.g. means) or other basic estimates (e.g. regression coefficient) AND variation (e.g. standard deviation) or associated estimates of uncertainty (e.g. confidence intervals)
[ ]	[x]	For null hypothesis testing, the test statistic (e.g. *F*, *t*, *r*) with confidence intervals, effect sizes, degrees of freedom and *P* value noted<br>*Give P values as exact values whenever suitable.*
[x]	[ ]	For Bayesian analysis, information on the choice of priors and Markov chain Monte Carlo settings
[x]	[ ]	For hierarchical and complex designs, identification of the appropriate level for tests and full reporting of outcomes
[x]	[ ]	Estimates of effect sizes (e.g. Cohen's *d*, Pearson's *r*), indicating how they were calculated

Our web collection on statistics for biologists contains articles on many of the points above.

Software and code

Policy information about availability of computer code

Data collection

Grip Strength: digital force gauge (Chatillon Force Measurement DFEII, Columbus Instruments)
in vivo gastrocnemius force: Aurora Scientific Mouse Muscle Physiology System (model 809B; Aurora Scientific, Inc., Aurora, Ontario, Canada). Forces were measured in mN using ASI 610A Dynamic Muscle Control v5.3 software.
Intact muscle mechanics: Aurora Scientific 1200A isolated muscle system using ASI 610A Dynamic Muscle Control v5.3 software.
Sarcomere length was collected by a high-speed camera and ASI 900B software (Aurora Scientific Inc. v4.196)
TEM and analysis: TECNAI Spirit G2 transmission electron microscope (FEI, Hillsboro, OR) with a side-mounted AMT Image Capture Engine V6.02 (4Mpix) digital camera, operated at 100 kV. ImageJ (v1.49, NIH, USA)
Fiber cross-sectional area analysis: Images were collected using an AxioCam MRc (Carl Zeiss) and analyzed with MATLAB (R2015b) application Semi-automatic Muscle Analysis using Segmentation of Histology (SMASH ver5).
SIM: Zeiss ELYRA S1 SR-SIM microscope was used with UV light and solid-state laser (405/488/561/642 nm) illumination sources, a 100× oil immersion objective (NA=1.46), and a sCMOS camera. Typical imaging was performed on a 49.34×49.34 !m2 area with 1280×1280 pixel dimensions. Typical image stacks comprising of 40 slices were acquired with 0.084!m Z-steps, five angles and five phases/angle for each slice. Image reconstruction and fluorescence intensity plot profile generation were performed with ZEN 2 software (Zeiss). Plot profiles of the antibody-labeled images were fit with Gaussian curves to determine the epitope peak position using Fityk 1.3.0 software.
X-ray diffraction: The images were analyzed using the MuscleX (v1.13.1) software developed at BioCAT (https://musclex.readthedocs.io/en/latest/. J. Jiratrakanvong, J. Shao, M. Menendez, X. Li, J. Li,Weikang Ma. G. Agam, T. Irving, MuscleX: software suite for diffraction X-ray imaging V1.13.1, doi:10.5281/zenodo.1195050, March 2018.)

Data analysis

Graphpad Prism 7

For manuscripts utilizing custom algorithms or software that are central to the research but not yet described in published literature, software must be made available to editors/reviewers. We strongly encourage code deposition in a community repository (e.g. GitHub). See the Nature Research guidelines for submitting code & software for further information.

Data

Policy information about availability of data

All manuscripts must include a data availability statement. This statement should provide the following information, where applicable:

- Accession codes, unique identifiers, or web links for publicly available datasets
- A list of figures that have associated raw data
- A description of any restrictions on data availability

All data are in the manuscript and attached as a source data file

Field-specific reporting

Please select the one below that is the best fit for your research. If you are not sure, read the appropriate sections before making your selection.

☒ Life sciences ☐ Behavioural & social sciences ☐ Ecological, evolutionary & environmental sciences

For a reference copy of the document with all sections, see nature.com/documents/nr-reporting-summary-flat.pdf

Life sciences study design

All studies must disclose on these points even when the disclosure is negative.

Sample size	The number of animals needed for the various experiments was based on power calculations, with the significance level set at p<0.01, and assuming variance similar to that of our previously published studies or present pilot studies. The assumed technical failure rate is based on previous experience and is as stated in the various aims. The method for calculating the number of required mice is according to Glantz and Slinker: Primer of applied regression and analysis of variance, McGraw-Hill, Inc. 2002.
Data exclusions	No data were excluded
Replication	Grip strength, in situ lower limb (foot plate) experiments and myosin heavy chain distribution (SDS-PAGE) was done at 3 time points showing similar results. X-ray diffraction was repeated at 2 different time points and with 2 different batches of animals. The results from X-ray experiments showed very similar reflection patterns. Multiple muscles (>=6) were examined from multiple (>=6) mice per group in the following experiments: Muscle weights (Figure 2), nebulin content (Figure 3) and myosin heavy chain distribution (Figure 4).
Randomization	N/A. All available mice of the correct genotypes and right age and gender were studied. There was no treatment performed and no need to subdivide the mice.
Blinding	We strictly applied blinding both during the experiments and during the data analysis.

Reporting for specific materials, systems and methods

We require information from authors about some types of materials, experimental systems and methods used in many studies. Here, indicate whether each material, system or method listed is relevant to your study. If you are not sure if a list item applies to your research, read the appropriate section before selecting a response.

Materials & experimental systems

n/a	Involved in the study	
☐	☒	Antibodies
☒	☐	Eukaryotic cell lines
☒	☐	Palaeontology
☐	☒	Animals and other organisms
☒	☐	Human research participants
☒	☐	Clinical data

Methods

n/a	Involved in the study	
☒	☐	ChIP-seq
☒	☐	Flow cytometry
☒	☐	MRI-based neuroimaging

Antibodies

Antibodies used	For cross-sectional analysis we used Laminin (1:400 rabbit L9393, Sigma-Aldrich), MHCI (1:75 IgG2b BA-F8, DSHB), MHCIIA (1:500 IgG1 SC-71, DSHB), MHCIIX exclusion (1:100 IgG1 BF-35, DSHB) and MHCIIB (1:50 IgM BF-F3, DSHB). The secondary antibodies were polyclonal Alexa Fluor 488-conjugated goat anti-rabbit [1:500 IgG (H+L) A11008, Thermo Fisher], polyclonal Alexa Fluor 350-conjugated goat anti-mouse [1:500 IgG2b A211440, Thermo Fisher], polyclonal Alexa Fluor 350-conjugated goat anti-mouse [1:500 IgG1 A21120, Thermo Fisher] and polyclonal Alexa Fluor 594-conjugated goat anti-mouse [1:500 IgM (Heavy Chain) A21044, Thermo Fisher].<br>For SR-SIM we used the following primary antibodies: a rabbit polyclonal anti-Tmod1 (3.33 ug/mL), rabbit polyclonal anti-ETmod

	(Tmod1, 3.33 ug/mL) and a mouse monoclonal anti-titin Ti102 (2.5 ug/mL). These antibodies are custom-made. Secondary antibodies included: AlexaFluor-647 conjugated donkey anti-rabbit IgG (1:200, ab150063, Abcam) and AlexaFluor-405 conjugated donkey anti-mouse IgG (1:200, ab175659, Invitrogen).
Validation	For verification of specificity and size of protein detected, Western blots were performed using as many controls as possible: bacterially-expressed protein fragments for the region containing the peptide-antigen, mutated protein that should not be recognized, WT cardiac and skeletal muscle tissue extracts, HOM tissue extracts from mice that have that region of the gene deleted. When cross-reactivity was observed, preabsorbing protocols were used to improve specificity.<br>Antibodies used for immunohistochemistry microscopy were screened for optimal dilution and detection method use wildtype or Hom (mice that have a deletion of that region of the gene) muscle tissues if possible. Secondary antibodies are purchased that have been preabsorbed against multiple mammalian species; no-primary-antibody controls are also used to verify specificity.<br>For Laminin (L9393, Sigma-Aldrich) independent validation - multiple antibodies against the target (Laminin) shows the same staining pattern. For MHCI (BA-F8, DHSB), MHCIIA (SC-71, DSHB), MHCIIX exclusion (BF-35, DSHB) and MHCIIB (BF-F3, DSHB) the staining pattern overlaps with expected myosin content, based on myosin heavy chain distribution on SDS-PAGE, in fast and slow twitch muscles considering genotypes.

Animals and other organisms

Policy information about studies involving animals; ARRIVE guidelines recommended for reporting animal research

Laboratory animals	Mice: C57BL/6J backcrossed for 10 generations. Grip strength was tested at 1, 4 and 10 months of age. In situ muscle function (foot plate) was performed at 3 (males) and 10 (females and males) months of age. Dissected at 4 and 10 months of age both females and males.
Wild animals	No wild animals were used in the study
Field-collected samples	No field studies were performed.
Ethics oversight	Ethical approval was granted by the University of Arizona and the Illinois Institute of Technology Institutional Animal Care and Use Committees and followed the NIH Guide for the Care and Use of Laboratory Animals.

Note that full information on the approval of the study protocol must also be provided in the manuscript.
